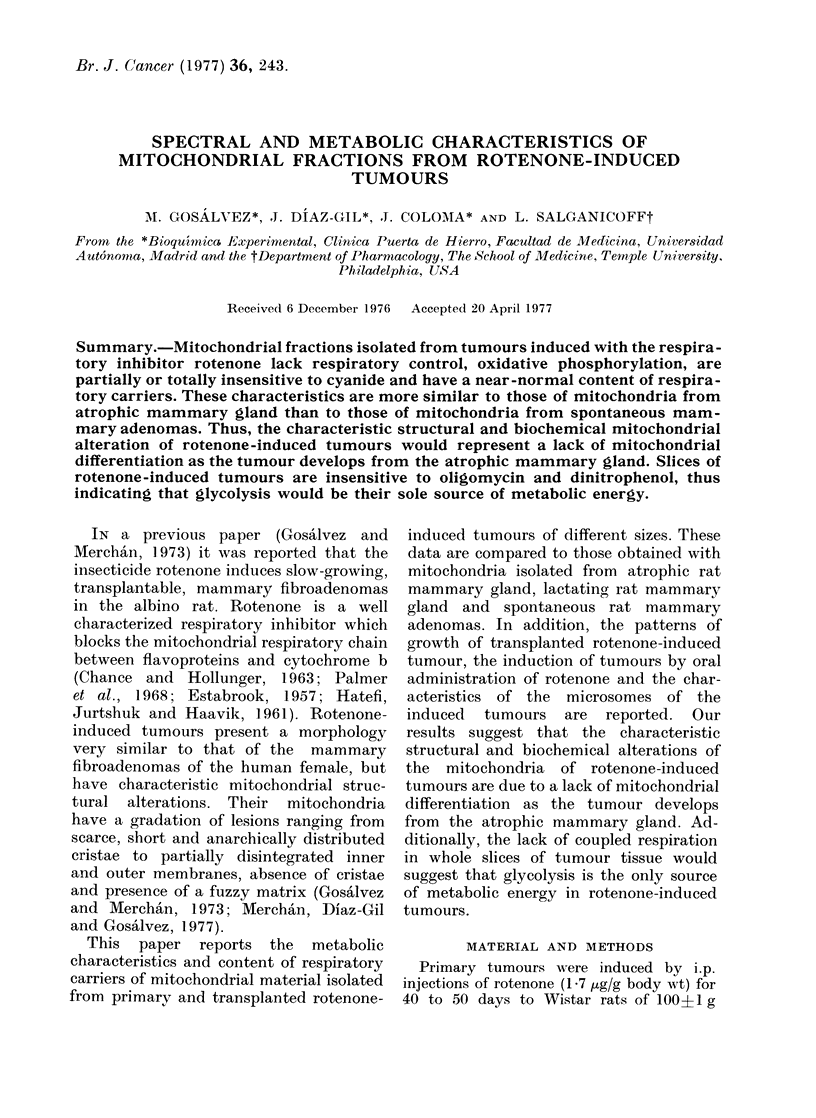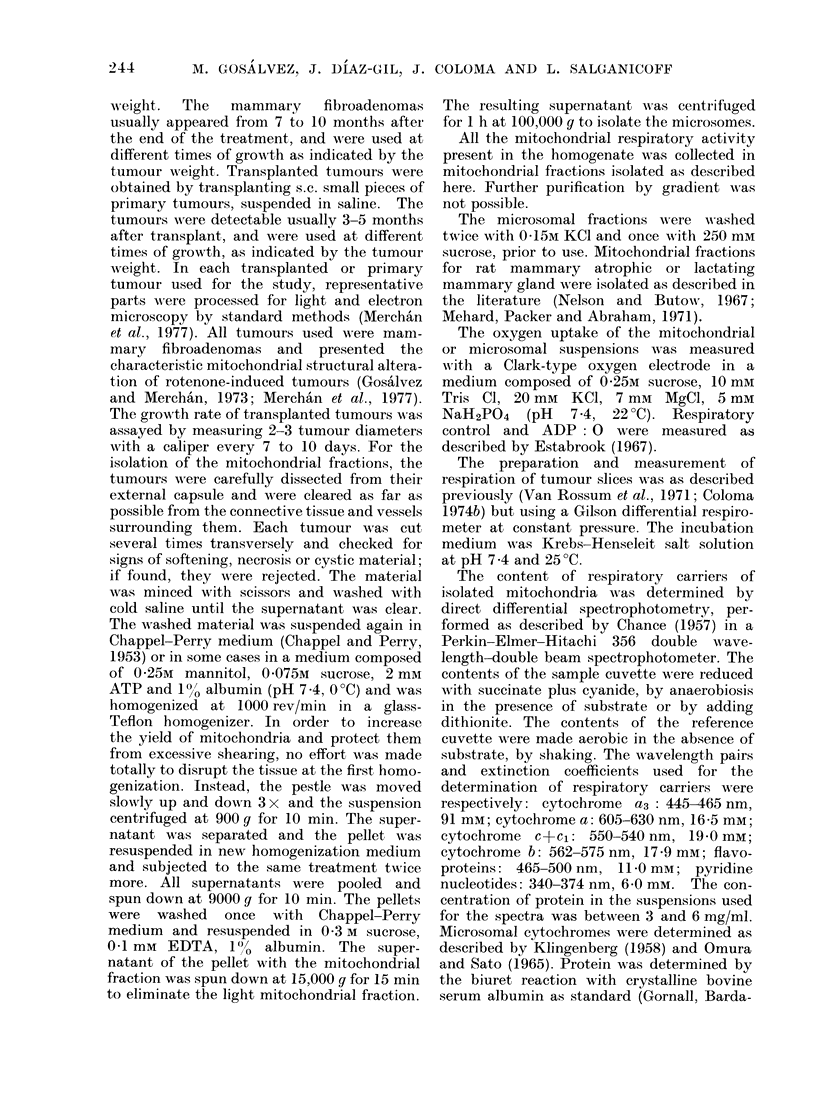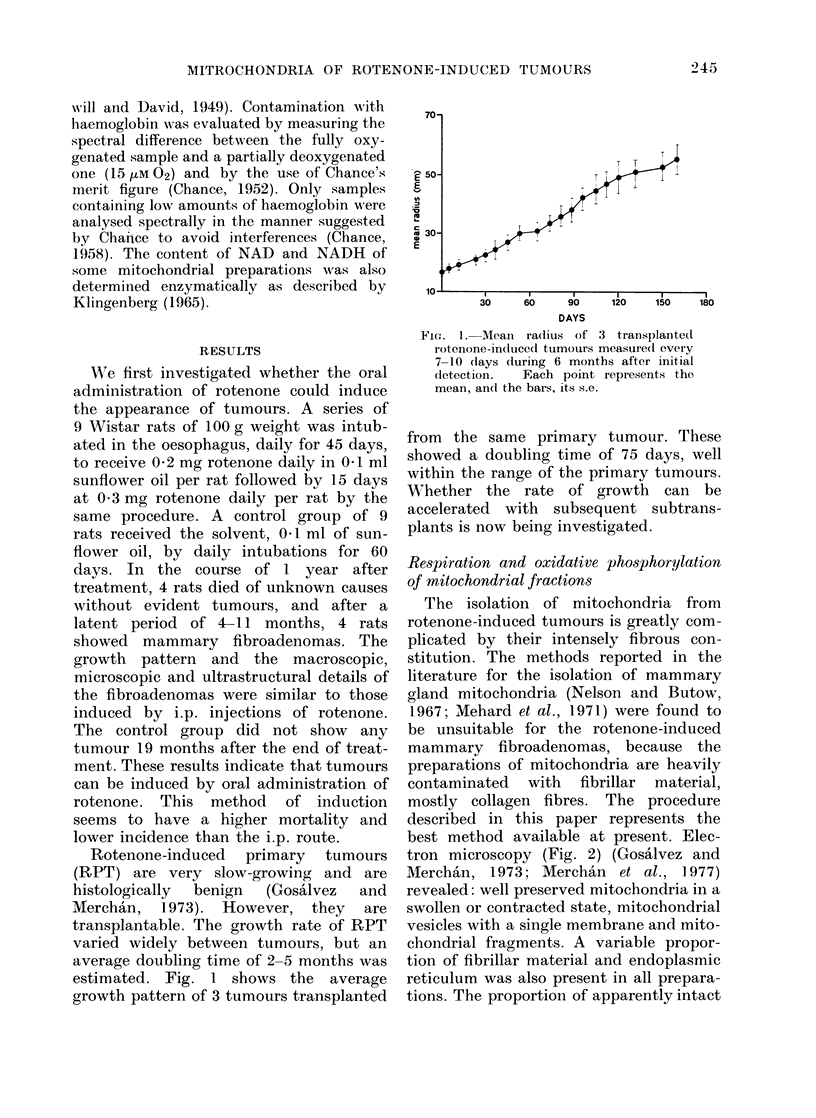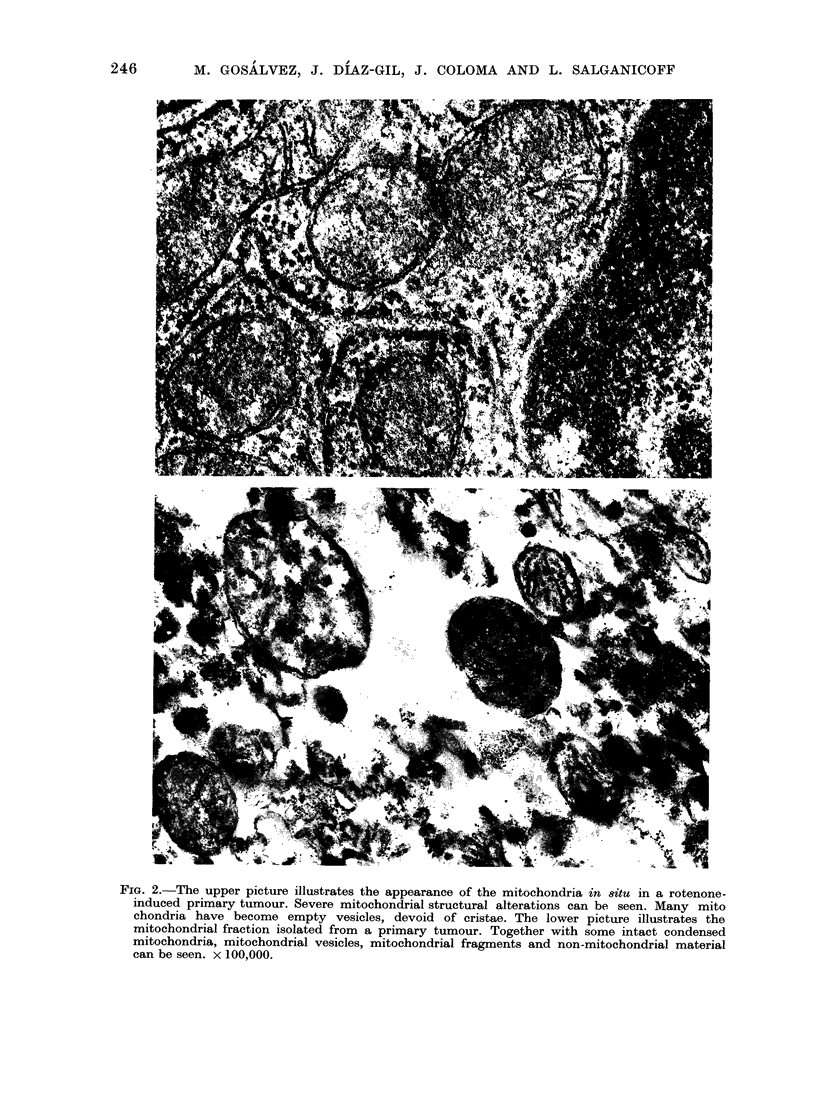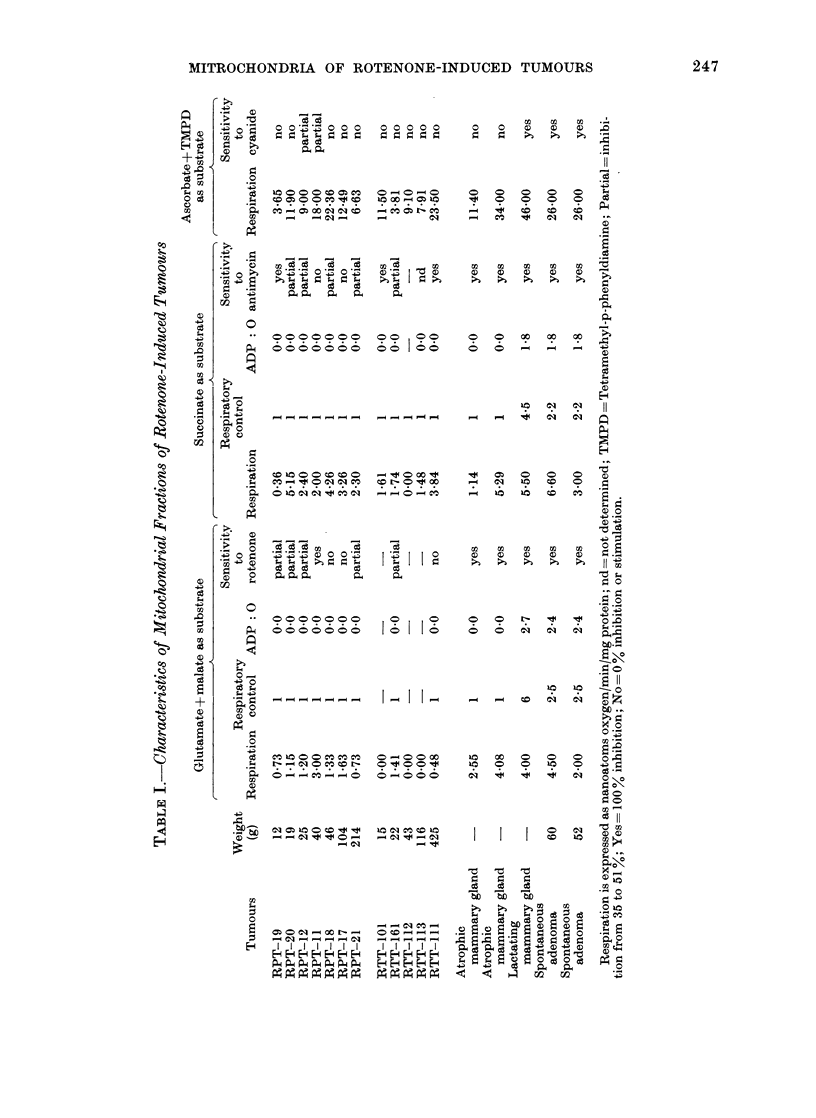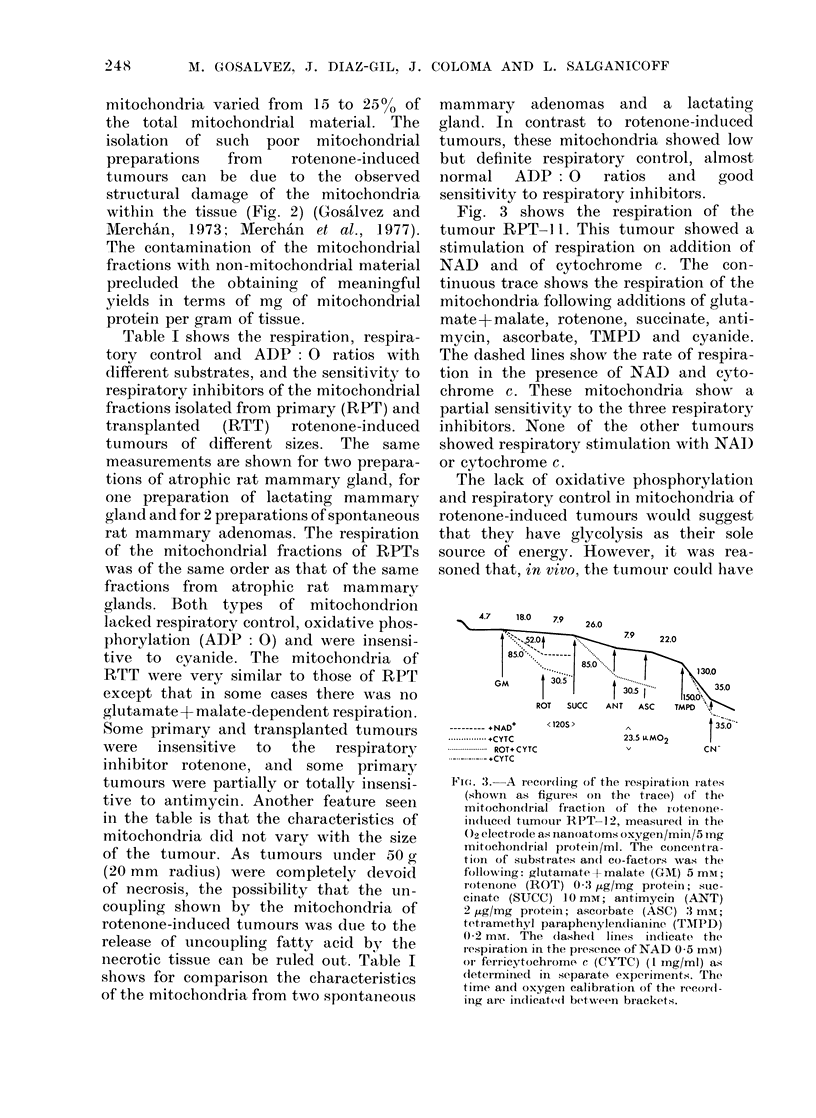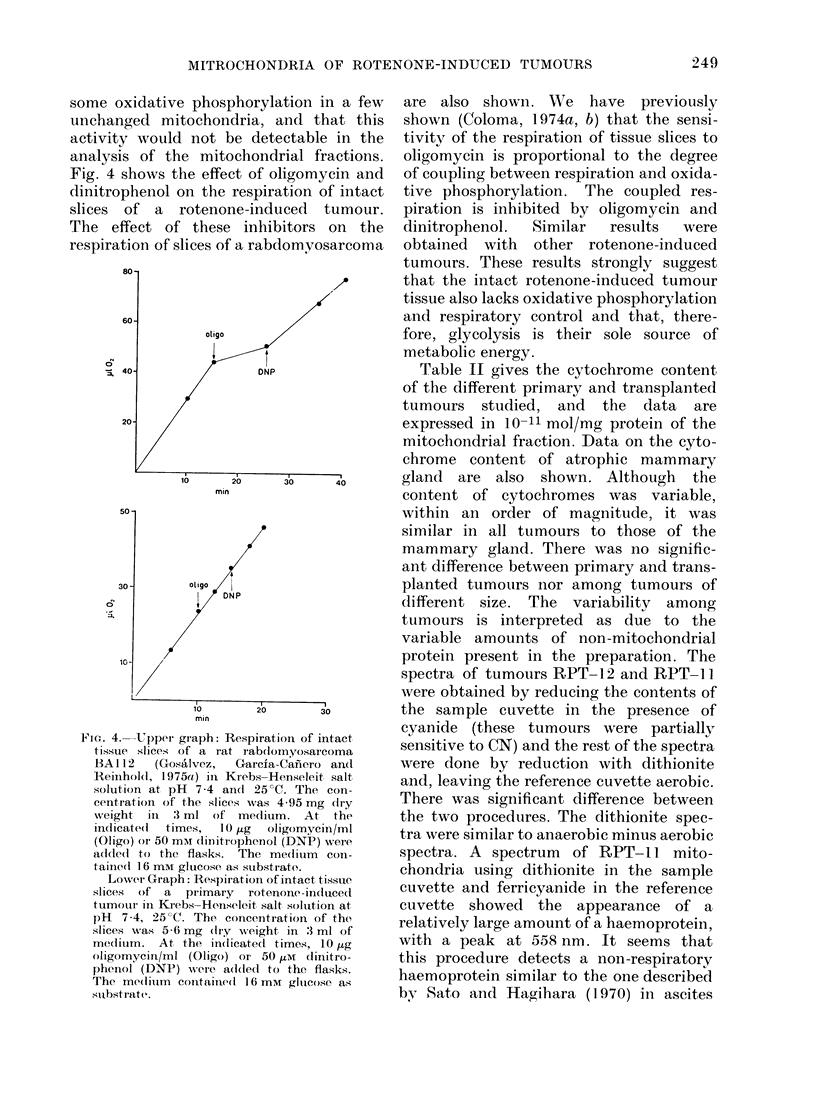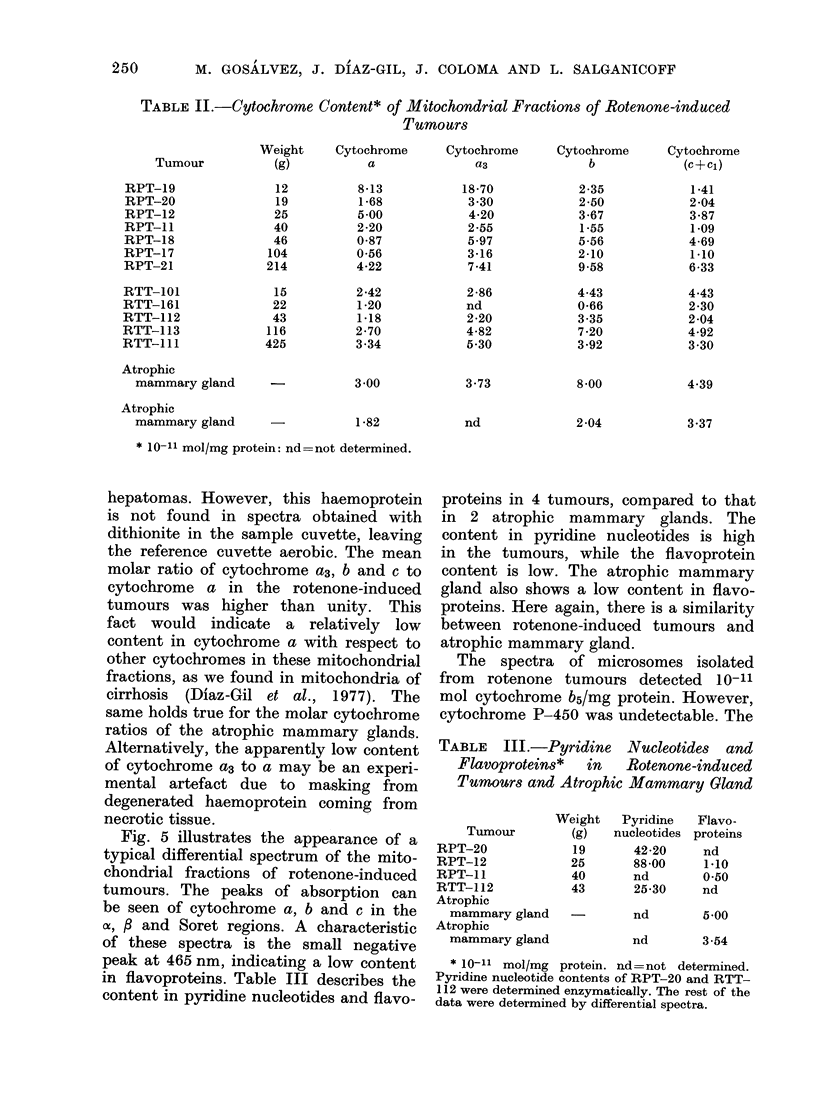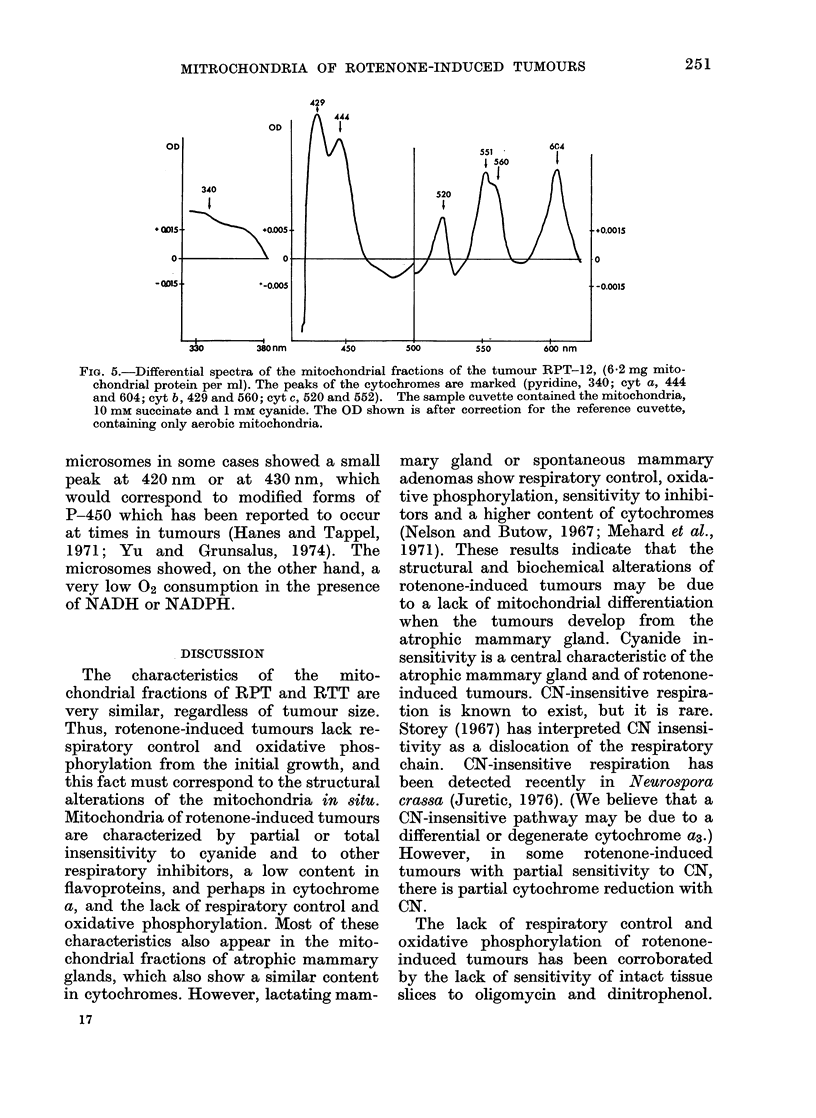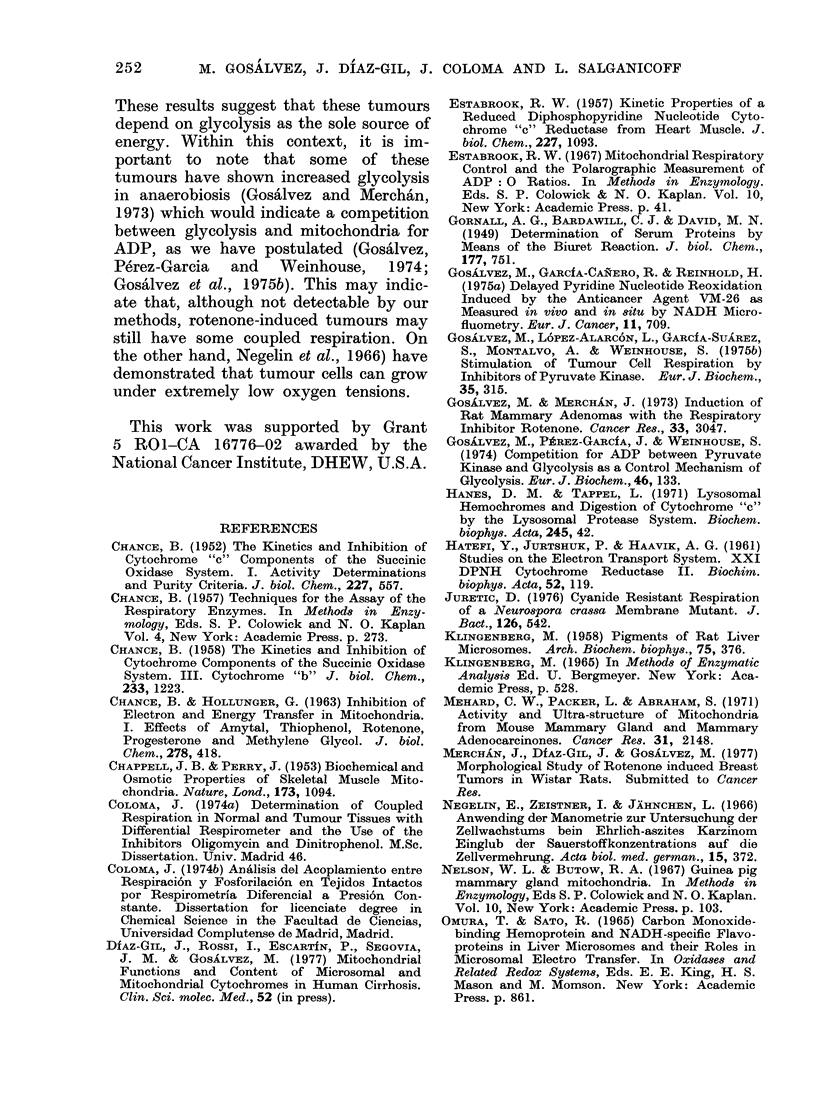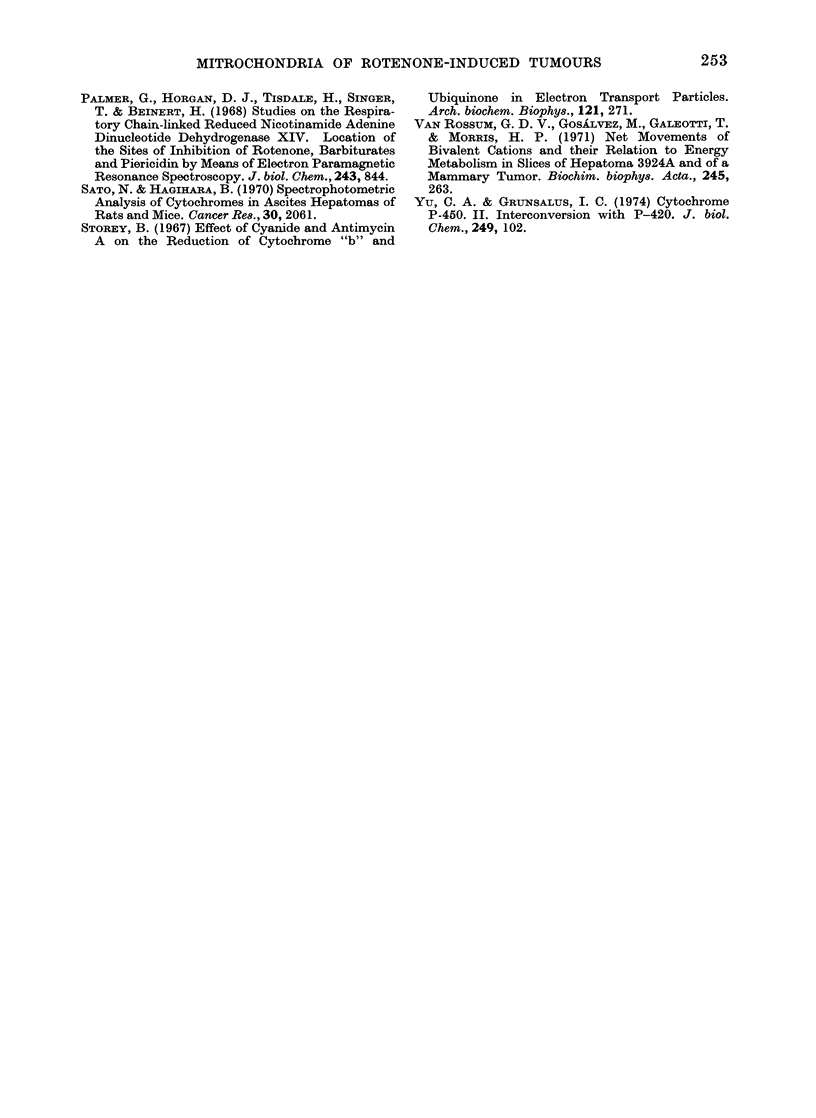# Spectral and metabolic characteristics of mitochondrial fractions from rotenone-induced tumours.

**DOI:** 10.1038/bjc.1977.184

**Published:** 1977-08

**Authors:** M. Gosálvez, J. Díaz-Gil, J. Coloma, L. Salganicoff

## Abstract

**Images:**


*Br. J. Cancer* (1977) **36**, 243.

### SPECTRAL AND METABOLIC CHARACTERISTICS OF MITOCHONDRIAL FRACTIONS FROM ROTENONE-INDUCED TUMOURS

M. GOSÁLVEZ*, J. DÍAZ-GIL*, J. COLOMA* AND L. SALGANICOFF†

*From the *Bioquímica Experimental, Clinica Puerta de Hierro, Facultad de Medicina, Universidad Autónoma, Madrid and the †Department of Pharmacology, The School of Medicine, Temple University, Philadelphia, USA*

Received 6 December 1976 Accepted 20 April 1977

**Summary.—Mitochondrial fractions isolated from tumours induced with the respiratory inhibitor rotenone lack respiratory control, oxidative phosphorylation, are partially or totally insensitive to cyanide and have a near-normal content of respiratory carriers. These characteristics are more similar to those of mitochondria from atrophic mammary gland than to those of mitochondria from spontaneous mammary adenomas. Thus, the characteristic structural and biochemical mitochondrial alteration of rotenone-induced tumours would represent a lack of mitochondrial differentiation as the tumour develops from the atrophic mammary gland. Slices of rotenone-induced tumours are insensitive to oligomycin and dinitrophenol, thus indicating that glycolysis would be their sole source of metabolic energy.**

IN a previous paper (Gosálvez and Merchán, 1973) it was reported that the insecticide rotenone induces slow-growing, transplantable, mammary fibroadenomas in the albino rat. Rotenone is a well characterized respiratory inhibitor which blocks the mitochondrial respiratory chain between flavoproteins and cytochrome b (Chance and Hollunger, 1963; Palmer *et al.*, 1968; Estabrook, 1957; Hatefi, Jurtshuk and Haavik, 1961). Rotenone-induced tumours present a morphology very similar to that of the mammary fibroadenomas of the human female, but have characteristic mitochondrial structural alterations. Their mitochondria have a gradation of lesions ranging from scarce, short and anarchically distributed cristae to partially disintegrated inner and outer membranes, absence of cristae and presence of a fuzzy matrix (Gosálvez and Merchán, 1973; Merchán, Díaz-Gil and Gosálvez, 1977).

This paper reports the metabolic characteristics and content of respiratory carriers of mitochondrial material isolated from primary and transplanted rotenone-induced tumours of different sizes. These data are compared to those obtained with mitochondria isolated from atrophic rat mammary gland, lactating rat mammary gland and spontaneous rat mammary adenomas. In addition, the patterns of growth of transplanted rotenone-induced tumour, the induction of tumours by oral administration of rotenone and the characteristics of the microsomes of the induced tumours are reported. Our results suggest that the characteristic structural and biochemical alterations of the mitochondria of rotenone-induced tumours are due to a lack of mitochondrial differentiation as the tumour develops from the atrophic mammary gland. Additionally, the lack of coupled respiration in whole slices of tumour tissue would suggest that glycolysis is the only source of metabolic energy in rotenone-induced tumours.

### MATERIAL AND METHODS

Primary tumours were induced by i.p. injections of rotenone (1·7 μg/g body wt) for 40 to 50 days to Wistar rats of 100±1 g weight. The mammary fibroadenomas usually appeared from 7 to 10 months after the end of the treatment, and were used at different times of growth as indicated by the tumour weight. Transplanted tumours were obtained by transplanting s.c. small pieces of primary tumours, suspended in saline. The tumours were detectable usually 3–5 months after transplant, and were used at different times of growth, as indicated by the tumour weight. In each transplanted or primary tumour used for the study, representative parts were processed for light and electron microscopy by standard methods (Merchán *et al.*, 1977). All tumours used were mammary fibroadenomas and presented the characteristic mitochondrial structural alteration of rotenone-induced tumours (Gosálvez and Merchán, 1973; Merchán *et al.*, 1977). The growth rate of transplanted tumours was assayed by measuring 2–3 tumour diameters with a caliper every 7 to 10 days. For the isolation of the mitochondrial fractions, the tumours were carefully dissected from their external capsule and were cleared as far as possible from the connective tissue and vessels surrounding them. Each tumour was cut several times transversely and checked for signs of softening, necrosis or cystic material; if found, they were rejected. The material was minced with scissors and washed with cold saline until the supernatant was clear. The washed material was suspended again in Chappel–Perry medium (Chappel and Perry, 1953) or in some cases in a medium composed of 0·25M mannitol, 0·075M sucrose, 2 mM ATP and 1% albumin (pH 7·4, 0 °C) and was homogenized at 1000 rev/min in a glass-Teflon homogenizer. In order to increase the yield of mitochondria and protect them from excessive shearing, no effort was made totally to disrupt the tissue at the first homogenization. Instead, the pestle was moved slowly up and down 3× and the suspension centrifuged at 900 *g* for 10 min. The supernatant was separated and the pellet was resuspended in new homogenization medium and subjected to the same treatment twice more. All supernatants were pooled and spun down at 9000 *g* for 10 min. The pellets were washed once with Chappel–Perry medium and resuspended in 0·3 M sucrose, 0·1 mM EDTA, 1% albumin. The supernatant of the pellet with the mitochondrial fraction was spun down at 15,000 *g* for 15 min to eliminate the light mitochondrial fraction. The resulting supernatant was centrifuged for 1 h at 100,000 *g* to isolate the microsomes.

All the mitochondrial respiratory activity present in the homogenate was collected in mitochondrial fractions isolated as described here. Further purification by gradient was not possible.

The microsomal fractions were washed twice with 0·15M KCl and once with 250 mM sucrose, prior to use. Mitochondrial fractions for rat mammary atrophic or lactating mammary gland were isolated as described in the literature (Nelson and Butow, 1967; Mehard, Packer and Abraham, 1971).

The oxygen uptake of the mitochondrial or microsomal suspensions was measured with a Clark-type oxygen electrode in a medium composed of 0·25M sucrose, 10 mM Tris Cl, 20 mM KCl, 7 mM MgCl, 5 mM $NaH_2PO_4$ (pH 7·4, 22 °C). Respiratory control and ADP : O were measured as described by Estabrook (1967).

The preparation and measurement of respiration of tumour slices was as described previously (Van Rossum *et al.*, 1971; Coloma 1974*b*) but using a Gilson differential respirometer at constant pressure. The incubation medium was Krebs–Henseleit salt solution at pH 7·4 and 25 °C.

The content of respiratory carriers of isolated mitochondria was determined by direct differential spectrophotometry, performed as described by Chance (1957) in a Perkin–Elmer–Hitachi 356 double wavelength–double beam spectrophotometer. The contents of the sample cuvette were reduced with succinate plus cyanide, by anaerobiosis in the presence of substrate or by adding dithionite. The contents of the reference cuvette were made aerobic in the absence of substrate, by shaking. The wavelength pairs and extinction coefficients used for the determination of respiratory carriers were respectively: cytochrome $a_3$ : 445–465 nm, 91 mM; cytochrome $a$: 605–630 nm, 16·5 mM; cytochrome $c+c_1$: 550–540 nm, 19·0 mM; cytochrome $b$: 562–575 nm, 17·9 mM; flavoproteins: 465–500 nm, 11·0 mM; pyridine nucleotides: 340–374 nm, 6·0 mM. The concentration of protein in the suspensions used for the spectra was between 3 and 6 mg/ml. Microsomal cytochromes were determined as described by Klingenberg (1958) and Omura and Sato (1965). Protein was determined by the biuret reaction with crystalline bovine serum albumin as standard (Gornall, Bardawill and David, 1949). Contamination with haemoglobin was evaluated by measuring the spectral difference between the fully oxygenated sample and a partially deoxygenated one (15 $\mu$M $O_2$) and by the use of Chance's merit figure (Chance, 1952). Only samples containing low amounts of haemoglobin were analysed spectrally in the manner suggested by Chance to avoid interferences (Chance, 1958). The content of NAD and NADH of some mitochondrial preparations was also determined enzymatically as described by Klingenberg (1965).

### RESULTS

We first investigated whether the oral administration of rotenone could induce the appearance of tumours. A series of 9 Wistar rats of 100 g weight was intubated in the oesophagus, daily for 45 days, to receive 0·2 mg rotenone daily in 0·1 ml sunflower oil per rat followed by 15 days at 0·3 mg rotenone daily per rat by the same procedure. A control group of 9 rats received the solvent, 0·1 ml of sunflower oil, by daily intubations for 60 days. In the course of 1 year after treatment, 4 rats died of unknown causes without evident tumours, and after a latent period of 4–11 months, 4 rats showed mammary fibroadenomas. The growth pattern and the macroscopic, microscopic and ultrastructural details of the fibroadenomas were similar to those induced by i.p. injections of rotenone. The control group did not show any tumour 19 months after the end of treatment. These results indicate that tumours can be induced by oral administration of rotenone. This method of induction seems to have a higher mortality and lower incidence than the i.p. route.

Rotenone-induced primary tumours (RPT) are very slow-growing and are histologically benign (Gosálvez and Merchán, 1973). However, they are transplantable. The growth rate of RPT varied widely between tumours, but an average doubling time of 2–5 months was estimated. Fig. 1 shows the average growth pattern of 3 tumours transplanted

Fig. 1.—Mean radius of 3 transplanted rotenone-induced tumours measured every 7–10 days during 6 months after initial detection. Each point represents the mean, and the bars, its s.e.

from the same primary tumour. These showed a doubling time of 75 days, well within the range of the primary tumours. Whether the rate of growth can be accelerated with subsequent subtransplants is now being investigated.

### *Respiration and oxidative phosphorylation of mitochondrial fractions*

The isolation of mitochondria from rotenone-induced tumours is greatly complicated by their intensely fibrous constitution. The methods reported in the literature for the isolation of mammary gland mitochondria (Nelson and Butow, 1967; Mehard *et al.*, 1971) were found to be unsuitable for the rotenone-induced mammary fibroadenomas, because the preparations of mitochondria are heavily contaminated with fibrillar material, mostly collagen fibres. The procedure described in this paper represents the best method available at present. Electron microscopy (Fig. 2) (Gosálvez and Merchán, 1973; Merchán *et al.*, 1977) revealed: well preserved mitochondria in a swollen or contracted state, mitochondrial vesicles with a single membrane and mitochondrial fragments. A variable proportion of fibrillar material and endoplasmic reticulum was also present in all preparations. The proportion of apparently intact

FIG. 2.—The upper picture illustrates the appearance of the mitochondria *in situ* in a rotenone-induced primary tumour. Severe mitochondrial structural alterations can be seen. Many mito chondria have become empty vesicles, devoid of cristae. The lower picture illustrates the mitochondrial fraction isolated from a primary tumour. Together with some intact condensed mitochondria, mitochondrial vesicles, mitochondrial fragments and non-mitochondrial material can be seen. $\times 100{,}000$.

TABLE I.—*Characteristics of Mitochondrial Fractions of Rotenone-Induced Tumours*

| Tumours | Weight (g) | Glutamate + malate as substrate: Respiration | Respiratory control | ADP : O | Sensitivity to rotenone | Succinate as substrate: Respiration | Respiratory control | ADP : O | Sensitivity to antimycin | Ascorbate + TMPD as substrate: Respiration | Sensitivity to cyanide |
|---|---|---|---|---|---|---|---|---|---|---|---|
| RPT–19 | 12 | 0·73 | 1 | 0·0 | partial | 0·36 | 1 | 0·0 | yes | 3·65 | no |
| RPT–20 | 19 | 1·15 | 1 | 0·0 | partial | 5·15 | 1 | 0·0 | partial | 11·90 | no |
| RPT–12 | 25 | 1·20 | 1 | 0·0 | partial | 2·40 | 1 | 0·0 | partial | 9·00 | partial |
| RPT–11 | 40 | 3·00 | 1 | 0·0 | yes | 2·00 | 1 | 0·0 | no | 18·00 | partial |
| RPT–18 | 46 | 1·33 | 1 | 0·0 | no | 4·26 | 1 | 0·0 | partial | 22·36 | no |
| RPT–17 | 104 | 1·63 | 1 | 0·0 | no | 3·26 | 1 | 0·0 | no | 12·49 | no |
| RPT–21 | 214 | 0·73 | 1 | 0·0 | partial | 2·30 | 1 | 0·0 | partial | 6·63 | no |
| RTT–101 | 15 | 0·00 | — | — | — | 1·61 | 1 | 0·0 | yes | 11·50 | no |
| RTT–161 | 22 | 1·41 | 1 | 0·0 | partial | 1·74 | 1 | 0·0 | partial | 3·81 | no |
| RTT–112 | 43 | 0·00 | — | — | — | 0·00 | 1 | — | — | 9·10 | no |
| RTT–113 | 116 | 0·00 | — | — | — | 1·48 | 1 | 0·0 | nd | 7·91 | no |
| RTT–111 | 425 | 0·48 | 1 | 0·0 | no | 3·84 | 1 | 0·0 | yes | 23·50 | no |
| Atrophic mammary gland | — | 2·55 | 1 | 0·0 | yes | 1·14 | 1 | 0·0 | yes | 11·40 | no |
| Atrophic mammary gland | — | 4·08 | 1 | 0·0 | yes | 5·29 | 1 | 0·0 | yes | 34·00 | no |
| Lactating mammary gland | — | 4·00 | 6 | 2·7 | yes | 5·50 | 4·5 | 1·8 | yes | 46·00 | yes |
| Spontaneous adenoma | 60 | 4·50 | 2·5 | 2·4 | yes | 6·60 | 2·2 | 1·8 | yes | 26·00 | yes |
| Spontaneous adenoma | 52 | 2·00 | 2·5 | 2·4 | yes | 3·00 | 2·2 | 1·8 | yes | 26·00 | yes |

Respiration is expressed as nanoatoms oxygen/min/mg protein; nd = not determined; TMPD = Tetramethyl-p-phenyldiamine; Partial = inhibition from 35 to 51%; Yes = 100% inhibition; No = 0% inhibition or stimulation.

mitochondria varied from 15 to 25% of the total mitochondrial material. The isolation of such poor mitochondrial preparations from rotenone-induced tumours can be due to the observed structural damage of the mitochondria within the tissue (Fig. 2) (Gosálvez and Merchán, 1973; Merchán *et al.*, 1977). The contamination of the mitochondrial fractions with non-mitochondrial material precluded the obtaining of meaningful yields in terms of mg of mitochondrial protein per gram of tissue.

Table I shows the respiration, respiratory control and ADP : O ratios with different substrates, and the sensitivity to respiratory inhibitors of the mitochondrial fractions isolated from primary (RPT) and transplanted (RTT) rotenone-induced tumours of different sizes. The same measurements are shown for two preparations of atrophic rat mammary gland, for one preparation of lactating mammary gland and for 2 preparations of spontaneous rat mammary adenomas. The respiration of the mitochondrial fractions of RPTs was of the same order as that of the same fractions from atrophic rat mammary glands. Both types of mitochondrion lacked respiratory control, oxidative phosphorylation (ADP : O) and were insensitive to cyanide. The mitochondria of RTT were very similar to those of RPT except that in some cases there was no glutamate+malate-dependent respiration. Some primary and transplanted tumours were insensitive to the respiratory inhibitor rotenone, and some primary tumours were partially or totally insensitive to antimycin. Another feature seen in the table is that the characteristics of mitochondria did not vary with the size of the tumour. As tumours under 50 g (20 mm radius) were completely devoid of necrosis, the possibility that the uncoupling shown by the mitochondria of rotenone-induced tumours was due to the release of uncoupling fatty acid by the necrotic tissue can be ruled out. Table I shows for comparison the characteristics of the mitochondria from two spontaneous mammary adenomas and a lactating gland. In contrast to rotenone-induced tumours, these mitochondria showed low but definite respiratory control, almost normal ADP : O ratios and good sensitivity to respiratory inhibitors.

Fig. 3 shows the respiration of the tumour RPT–11. This tumour showed a stimulation of respiration on addition of NAD and of cytochrome *c*. The continuous trace shows the respiration of the mitochondria following additions of glutamate+malate, rotenone, succinate, antimycin, ascorbate, TMPD and cyanide. The dashed lines show the rate of respiration in the presence of NAD and cytochrome *c*. These mitochondria show a partial sensitivity to the three respiratory inhibitors. None of the other tumours showed respiratory stimulation with NAD or cytochrome *c*.

The lack of oxidative phosphorylation and respiratory control in mitochondria of rotenone-induced tumours would suggest that they have glycolysis as their sole source of energy. However, it was reasoned that, *in vivo*, the tumour could have

FIG. 3.—A recording of the respiration rates (shown as figures on the trace) of the mitochondrial fraction of the rotenone-induced tumour RPT–12, measured in the $O_2$ electrode as nanoatoms oxygen/min/5 mg mitochondrial protein/ml. The concentration of substrates and co-factors was the following: glutamate+malate (GM) 5 mM; rotenone (ROT) 0·3 μg/mg protein; succinate (SUCC) 10 mM; antimycin (ANT) 2 μg/mg protein; ascorbate (ASC) 3 mM; tetramethyl paraphenylendianine (TMPD) 0·2 mM. The dashed lines indicate the respiration in the presence of NAD 0·5 mM) or ferricytochrome *c* (CYTC) (1 mg/ml) as determined in separate experiments. The time and oxygen calibration of the recording are indicated between brackets.

some oxidative phosphorylation in a few unchanged mitochondria, and that this activity would not be detectable in the analysis of the mitochondrial fractions. Fig. 4 shows the effect of oligomycin and dinitrophenol on the respiration of intact slices of a rotenone-induced tumour. The effect of these inhibitors on the respiration of slices of a rabdomyosarcoma

FIG. 4.—Upper graph: Respiration of intact tissue slices of a rat rabdomyosarcoma BA112 (Gosálvez, García-Cañero and Reinhold, 1975*a*) in Krebs–Henseleit salt solution at pH 7·4 and 25 °C. The concentration of the slices was 4·95 mg dry weight in 3 ml of medium. At the indicated times, 10 μg oligomycin/ml (Oligo) or 50 mM dinitrophenol (DNP) were added to the flasks. The medium contained 16 mM glucose as substrate.

Lower Graph: Respiration of intact tissue slices of a primary rotenone-induced tumour in Krebs–Henseleit salt solution at pH 7·4, 25 °C. The concentration of the slices was 5·6 mg dry weight in 3 ml of medium. At the indicated times, 10 μg oligomycin/ml (Oligo) or 50 μM dinitrophenol (DNP) were added to the flasks. The medium contained 16 mM glucose as substrate.

are also shown. We have previously shown (Coloma, 1974*a*, *b*) that the sensitivity of the respiration of tissue slices to oligomycin is proportional to the degree of coupling between respiration and oxidative phosphorylation. The coupled respiration is inhibited by oligomycin and dinitrophenol. Similar results were obtained with other rotenone-induced tumours. These results strongly suggest that the intact rotenone-induced tumour tissue also lacks oxidative phosphorylation and respiratory control and that, therefore, glycolysis is their sole source of metabolic energy.

Table II gives the cytochrome content of the different primary and transplanted tumours studied, and the data are expressed in $10^{-11}$ mol/mg protein of the mitochondrial fraction. Data on the cytochrome content of atrophic mammary gland are also shown. Although the content of cytochromes was variable, within an order of magnitude, it was similar in all tumours to those of the mammary gland. There was no significant difference between primary and transplanted tumours nor among tumours of different size. The variability among tumours is interpreted as due to the variable amounts of non-mitochondrial protein present in the preparation. The spectra of tumours RPT–12 and RPT–11 were obtained by reducing the contents of the sample cuvette in the presence of cyanide (these tumours were partially sensitive to CN) and the rest of the spectra were done by reduction with dithionite and, leaving the reference cuvette aerobic. There was significant difference between the two procedures. The dithionite spectra were similar to anaerobic minus aerobic spectra. A spectrum of RPT–11 mitochondria using dithionite in the sample cuvette and ferricyanide in the reference cuvette showed the appearance of a relatively large amount of a haemoprotein, with a peak at 558 nm. It seems that this procedure detects a non-respiratory haemoprotein similar to the one described by Sato and Hagihara (1970) in ascites

TABLE II.—*Cytochrome Content* of Mitochondrial Fractions of Rotenone-induced Tumours*

| Tumour | Weight (g) | Cytochrome $a$ | Cytochrome $a_3$ | Cytochrome $b$ | Cytochrome $(c+c_1)$ |
|---|---|---|---|---|---|
| RPT–19 | 12 | 8·13 | 18·70 | 2·35 | 1·41 |
| RPT–20 | 19 | 1·68 | 3·30 | 2·50 | 2·04 |
| RPT–12 | 25 | 5·00 | 4·20 | 3·67 | 3·87 |
| RPT–11 | 40 | 2·20 | 2·55 | 1·55 | 1·09 |
| RPT–18 | 46 | 0·87 | 5·97 | 5·56 | 4·69 |
| RPT–17 | 104 | 0·56 | 3·16 | 2·10 | 1·10 |
| RPT–21 | 214 | 4·22 | 7·41 | 9·58 | 6·33 |
| RTT–101 | 15 | 2·42 | 2·86 | 4·43 | 4·43 |
| RTT–161 | 22 | 1·20 | nd | 0·66 | 2·30 |
| RTT–112 | 43 | 1·18 | 2·20 | 3·35 | 2·04 |
| RTT–113 | 116 | 2·70 | 4·82 | 7·20 | 4·92 |
| RTT–111 | 425 | 3·34 | 5·30 | 3·92 | 3·30 |
| Atrophic mammary gland | — | 3·00 | 3·73 | 8·00 | 4·39 |
| Atrophic mammary gland | — | 1·82 | nd | 2·04 | 3·37 |

* $10^{-11}$ mol/mg protein: nd=not determined.

hepatomas. However, this haemoprotein is not found in spectra obtained with dithionite in the sample cuvette, leaving the reference cuvette aerobic. The mean molar ratio of cytochrome $a_3$, $b$ and $c$ to cytochrome $a$ in the rotenone-induced tumours was higher than unity. This fact would indicate a relatively low content in cytochrome $a$ with respect to other cytochromes in these mitochondrial fractions, as we found in mitochondria of cirrhosis (Díaz-Gil *et al.*, 1977). The same holds true for the molar cytochrome ratios of the atrophic mammary glands. Alternatively, the apparently low content of cytochrome $a_3$ to $a$ may be an experimental artefact due to masking from degenerated haemoprotein coming from necrotic tissue.

Fig. 5 illustrates the appearance of a typical differential spectrum of the mitochondrial fractions of rotenone-induced tumours. The peaks of absorption can be seen of cytochrome $a$, $b$ and $c$ in the $\alpha$, $\beta$ and Soret regions. A characteristic of these spectra is the small negative peak at 465 nm, indicating a low content in flavoproteins. Table III describes the content in pyridine nucleotides and flavoproteins in 4 tumours, compared to that in 2 atrophic mammary glands. The content in pyridine nucleotides is high in the tumours, while the flavoprotein content is low. The atrophic mammary gland also shows a low content in flavoproteins. Here again, there is a similarity between rotenone-induced tumours and atrophic mammary gland.

The spectra of microsomes isolated from rotenone tumours detected $10^{-11}$ mol cytochrome $b_5$/mg protein. However, cytochrome P–450 was undetectable. The

TABLE III.—*Pyridine Nucleotides and Flavoproteins* in Rotenone-induced Tumours and Atrophic Mammary Gland*

| Tumour | Weight (g) | Pyridine nucleotides | Flavo-proteins |
|---|---|---|---|
| RPT–20 | 19 | 42·20 | nd |
| RPT–12 | 25 | 88·00 | 1·10 |
| RPT–11 | 40 | nd | 0·50 |
| RTT–112 | 43 | 25·30 | nd |
| Atrophic mammary gland | — | nd | 5·00 |
| Atrophic mammary gland | | nd | 3·54 |

* $10^{-11}$ mol/mg protein. nd=not determined. Pyridine nucleotide contents of RPT–20 and RTT–112 were determined enzymatically. The rest of the data were determined by differential spectra.

FIG. 5.—Differential spectra of the mitochondrial fractions of the tumour RPT–12, (6·2 mg mitochondrial protein per ml). The peaks of the cytochromes are marked (pyridine, 340; cyt *a*, 444 and 604; cyt *b*, 429 and 560; cyt *c*, 520 and 552). The sample cuvette contained the mitochondria, 10 mM succinate and 1 mM cyanide. The OD shown is after correction for the reference cuvette, containing only aerobic mitochondria.

microsomes in some cases showed a small peak at 420 nm or at 430 nm, which would correspond to modified forms of P–450 which has been reported to occur at times in tumours (Hanes and Tappel, 1971; Yu and Grunsalus, 1974). The microsomes showed, on the other hand, a very low $O_2$ consumption in the presence of NADH or NADPH.

### DISCUSSION

The characteristics of the mitochondrial fractions of RPT and RTT are very similar, regardless of tumour size. Thus, rotenone-induced tumours lack respiratory control and oxidative phosphorylation from the initial growth, and this fact must correspond to the structural alterations of the mitochondria *in situ*. Mitochondria of rotenone-induced tumours are characterized by partial or total insensitivity to cyanide and to other respiratory inhibitors, a low content in flavoproteins, and perhaps in cytochrome *a*, and the lack of respiratory control and oxidative phosphorylation. Most of these characteristics also appear in the mitochondrial fractions of atrophic mammary glands, which also show a similar content in cytochromes. However, lactating mammary gland or spontaneous mammary adenomas show respiratory control, oxidative phosphorylation, sensitivity to inhibitors and a higher content of cytochromes (Nelson and Butow, 1967; Mehard *et al.*, 1971). These results indicate that the structural and biochemical alterations of rotenone-induced tumours may be due to a lack of mitochondrial differentiation when the tumours develop from the atrophic mammary gland. Cyanide insensitivity is a central characteristic of the atrophic mammary gland and of rotenone-induced tumours. CN-insensitive respiration is known to exist, but it is rare. Storey (1967) has interpreted CN insensitivity as a dislocation of the respiratory chain. CN-insensitive respiration has been detected recently in *Neurospora crassa* (Juretic, 1976). (We believe that a CN-insensitive pathway may be due to a differential or degenerate cytochrome $a_3$.) However, in some rotenone-induced tumours with partial sensitivity to CN, there is partial cytochrome reduction with CN.

The lack of respiratory control and oxidative phosphorylation of rotenone-induced tumours has been corroborated by the lack of sensitivity of intact tissue slices to oligomycin and dinitrophenol.

These results suggest that these tumours depend on glycolysis as the sole source of energy. Within this context, it is important to note that some of these tumours have shown increased glycolysis in anaerobiosis (Gosálvez and Merchán, 1973) which would indicate a competition between glycolysis and mitochondria for ADP, as we have postulated (Gosálvez, Pérez-Garcia and Weinhouse, 1974; Gosálvez *et al.*, 1975*b*). This may indicate that, although not detectable by our methods, rotenone-induced tumours may still have some coupled respiration. On the other hand, Negelin *et al.*, 1966) have demonstrated that tumour cells can grow under extremely low oxygen tensions.

This work was supported by Grant 5 RO1-CA 16776-02 awarded by the National Cancer Institute, DHEW, U.S.A.

### REFERENCES

CHANCE, B. (1952) The Kinetics and Inhibition of Cytochrome "c" Components of the Succinic Oxidase System. I. Activity Determinations and Purity Criteria. *J. biol. Chem.*, **227**, 557.

CHANCE, B. (1957) Techniques for the Assay of the Respiratory Enzymes. In *Methods in Enzymology*, Eds. S. P. Colowick and N. O. Kaplan Vol. 4, New York: Academic Press. p. 273.

CHANCE, B. (1958) The Kinetics and Inhibition of Cytochrome Components of the Succinic Oxidase System. III. Cytochrome "b" *J. biol. Chem.*, **233**, 1223.

CHANCE, B. & HOLLUNGER, G. (1963) Inhibition of Electron and Energy Transfer in Mitochondria. I. Effects of Amytal, Thiophenol, Rotenone, Progesterone and Methylene Glycol. *J. biol. Chem.*, **278**, 418.

CHAPPELL, J. B. & PERRY, J. (1953) Biochemical and Osmotic Properties of Skeletal Muscle Mitochondria. *Nature, Lond.*, **173**, 1094.

COLOMA, J. (1974*a*) Determination of Coupled Respiration in Normal and Tumour Tissues with Differential Respirometer and the Use of the Inhibitors Oligomycin and Dinitrophenol. M.Sc. Dissertation. Univ. Madrid 46.

COLOMA, J. (1974*b*) Análisis del Acoplamiento entre Respiración y Fosforilación en Tejidos Intactos por Respirometría Diferencial a Presión Constante. Dissertation for licenciate degree in Chemical Science in the Facultad de Ciencias, Universidad Complutense de Madrid, Madrid.

DÍAZ-GIL, J., ROSSI, I., ESCARTÍN, P., SEGOVIA, J. M. & GOSÁLVEZ, M. (1977) Mitochondrial Functions and Content of Microsomal and Mitochondrial Cytochromes in Human Cirrhosis. *Clin. Sci. molec. Med.*, **52** (in press).

ESTABROOK, R. W. (1957) Kinetic Properties of a Reduced Diphosphopyridine Nucleotide Cytochrome "c" Reductase from Heart Muscle. *J. biol. Chem.*, **227**, 1093.

ESTABROOK, R. W. (1967) Mitochondrial Respiratory Control and the Polarographic Measurement of ADP : O Ratios. In *Methods in Enzymology*. Eds. S. P. Colowick & N. O. Kaplan. Vol. 10, New York: Academic Press. p. 41.

GORNALL, A. G., BARDAWILL, C. J. & DAVID, M. N. (1949) Determination of Serum Proteins by Means of the Biuret Reaction. *J. biol. Chem.*, **177**, 751.

GOSÁLVEZ, M., GARCÍA-CAÑERO, R. & REINHOLD, H. (1975*a*) Delayed Pyridine Nucleotide Reoxidation Induced by the Anticancer Agent VM-26 as Measured *in vivo* and *in situ* by NADH Microfluometry. *Eur. J. Cancer*, **11**, 709.

GOSÁLVEZ, M., LÓPEZ-ALARCÓN, L., GARCÍA-SUÁREZ, S., MONTALVO, A. & WEINHOUSE, S. (1975*b*) Stimulation of Tumour Cell Respiration by Inhibitors of Pyruvate Kinase. *Eur. J. Biochem.*, **35**, 315.

GOSÁLVEZ, M. & MERCHÁN, J. (1973) Induction of Rat Mammary Adenomas with the Respiratory Inhibitor Rotenone. *Cancer Res.*, **33**, 3047.

GOSÁLVEZ, M., PÉREZ-GARCÍA, J. & WEINHOUSE, S. (1974) Competition for ADP between Pyruvate Kinase and Glycolysis as a Control Mechanism of Glycolysis. *Eur. J. Biochem.*, **46**, 133.

HANES, D. M. & TAPPEL, L. (1971) Lysosomal Hemochromes and Digestion of Cytochrome "c" by the Lysosomal Protease System. *Biochem. biophys. Acta*, **245**, 42.

HATEFI, Y., JURTSHUK, P. & HAAVIK, A. G. (1961) Studies on the Electron Transport System. XXI DPNH Cytochrome Reductase II. *Biochim. biophys. Acta*, **52**, 119.

JURETIC, D. (1976) Cyanide Resistant Respiration of a *Neurospora crassa* Membrane Mutant. *J. Bact.*, **126**, 542.

KLINGENBERG, M. (1958) Pigments of Rat Liver Microsomes. *Arch. Biochem. biophys.*, **75**, 376.

KLINGENBERG, M. (1965) In *Methods of Enzymatic Analysis* Ed. U. Bergmeyer. New York: Academic Press, p. 528.

MEHARD, C. W., PACKER, L. & ABRAHAM, S. (1971) Activity and Ultra-structure of Mitochondria from Mouse Mammary Gland and Mammary Adenocarcinones. *Cancer Res.* **31**, 2148.

MERCHÁN, J., DÍAZ-GIL, J. & GOSÁLVEZ, M. (1977) Morphological Study of Rotenone induced Breast Tumors in Wistar Rats. Submitted to *Cancer Res.*

NEGELIN, E., ZEISTNER, I. & JÄHNCHEN, L. (1966) Anwending der Manometrie zur Untersuchung der Zellwachstums bein Ehrlich-aszites Karzinom Einglub der Sauerstoffkonzentrations auf die Zellvermehrung. *Acta biol. med. german.*, **15**, 372.

NELSON, W. L. & BUTOW, R. A. (1967) Guinea pig mammary gland mitochondria. In *Methods in Enzymology*, Eds S. P. Colowick and N. O. Kaplan. Vol. 10, New York: Academic Press. p. 103.

OMURA, T. & SATO, R. (1965) Carbon Monoxide-binding Hemoprotein and NADH-specific Flavoproteins in Liver Microsomes and their Roles in Microsomal Electro Transfer. In *Oxidases and Related Redox Systems*, Eds. E. E. King, H. S. Mason and M. Momson. New York: Academic Press. p. 861.

Palmer, G., Horgan, D. J., Tisdale, H., Singer, T. & Beinert, H. (1968) Studies on the Respiratory Chain-linked Reduced Nicotinamide Adenine Dinucleotide Dehydrogenase XIV. Location of the Sites of Inhibition of Rotenone, Barbiturates and Piericidin by Means of Electron Paramagnetic Resonance Spectroscopy. *J. biol. Chem.*, **243**, 844.

Sato, N. & Hagihara, B. (1970) Spectrophotometric Analysis of Cytochromes in Ascites Hepatomas of Rats and Mice. *Cancer Res.*, **30**, 2061.

Storey, B. (1967) Effect of Cyanide and Antimycin A on the Reduction of Cytochrome "b" and Ubiquinone in Electron Transport Particles. *Arch. biochem. Biophys.*, **121**, 271.

Van Rossum, G. D. V., Gosálvez, M., Galeotti, T. & Morris, H. P. (1971) Net Movements of Bivalent Cations and their Relation to Energy Metabolism in Slices of Hepatoma 3924A and of a Mammary Tumor. *Biochim. biophys. Acta.*, **245**, 263.

Yu, C. A. & Grunsalus, I. C. (1974) Cytochrome P-450. II. Interconversion with P–420. *J. biol. Chem.*, **249**, 102.